# MAPLE-Deposited Perylene Diimide Derivative Based Layers for Optoelectronic Applications

**DOI:** 10.3390/nano14211733

**Published:** 2024-10-29

**Authors:** Carmen Breazu, Mihaela Girtan, Anca Stanculescu, Nicoleta Preda, Oana Rasoga, Andreea Costas, Ana Maria Catargiu, Gabriel Socol, Andrei Stochioiu, Gianina Popescu-Pelin, Sorina Iftimie, Gabriela Petre, Marcela Socol

**Affiliations:** 1 National Institute of Materials Physics, 405A Atomistilor Street, 077125 Magurele, Romania; 2 Laboratoire LPHIA, Université d’Angers, LUNAM, 2 Bd. Lavoisier, 49045 Angers, France; 3 P. Poni Institute of Macromolecular Chemistry, 41 A Gr. Ghica Voda Alley, 700487 Iasi, Romania; 4 National Institute for Lasers, Plasma and Radiation Physics, 409 Atomistilor Street, 077125 Magurele, Romania; 5 Faculty of Physics, University of Bucharest, 405 Atomistilor Street, 077125 Magurele, Romania

**Keywords:** MAPLE, perylene diimide derivative, OPV, BHJ, fullerene

## Abstract

Nowadays, the development of devices based on organic materials is an interesting research challenge. The performance of such devices is strongly influenced by material selection, material properties, design, and the manufacturing process. Usually, buckminsterfullerene (C60) is employed as electron transport material in organic photovoltaic (OPV) devices due to its high mobility. However, considering its low solubility, there have been many attempts to replace it with more soluble non-fullerene compounds. In this study, bulk heterojunction thin films with various compositions of zinc phthalocyanine (ZnPc), a perylene diimide derivative, or C60 were prepared by matrix-assisted pulsed laser evaporation (MAPLE) technique to assess the influence of C60 replacement on fabricated heterostructure properties. The investigations revealed that the optical features and the electrical parameters of the organic heterostructures based on this perylene diimide derivative used as an organic acceptor were improved. An increase in the J_SC_ value (4.3 × 10^−4^ A/cm^2^) was obtained for the structures where the perylene diimide derivative acceptor entirely replaced C60 compared to the J_SC_ value (7.5 × 10^−8^ A/cm^2^) for the heterostructure fabricated only with fullerene. These results are encouraging, demonstrating the potential of non-fullerene compounds as electron transport material in OPV devices.

## 1. Introduction

In the last decade, various renewable energy sources have been considered due to the growing demand for clean energy [1]. Solar energy can provide the daily energy requirement if efficient collection of the sun’s rays is accomplished. A large variety of materials/compounds have been studied for integration in optoelectronic devices [2,3,4,5,6,7,8]. Today, in photovoltaic systems, organic materials are in the spotlight due to their properties such as flexibility, high absorption coefficients, lightweight, transparency, and unexpected efficiencies (over 19%) [2,3,9,10]. Organic-based devices have been successfully applied in building-integrated photovoltaics (roofs and walls) and wearable electronics (clothing, bags), etc. [2,3,11].

Although the efficiencies of organic photovoltaic (OPV) cells cannot yet compete with inorganic-based photovoltaic cells, different approaches to improve their power conversion efficiency (PCE) are being sought. It is known that higher PCE values can be achieved by enhancing open-circuit voltage (*V*_oc_), short-circuit current density (*J*_sc_), and fill factor (FF) parameters, which depend on: (i) photon absorption and exciton generation efficiency, (ii) exciton diffusion and dissociation efficiency, and (iii) charge transport and collection efficiency [3,10].

The poor coverage of the solar spectrum by organic semiconductors due to their narrow intrinsic absorption bands can be one of the reasons for low OPV cell efficiencies [12]. On the other hand, the poor charge carrier mobility of organic semiconducting materials and the reduced exciton diffusion length limit the thickness of the active layer to around 100 nm [13]. However, reduced thickness does not ensure efficient collection of the incident light and the number of excitons that further must dissociate in free charge carriers is reduced, limiting device performance.

Many strategies have been implemented to enhance the performance of OPV cells [2,3,9,14]. Bulk heterojunction configuration (BHJ) is considered an efficient pathway for obtaining high-performance photovoltaics due to the interpenetrating network formed between donor and acceptor, which facilitates exciton dissociation and charge carrier transport [15]. Another approach involves the use of ternary blends to produce active layers [16]. Compared to its binary counterpart, a ternary blend solar cell has an additional electron donor or acceptor organic compound, generally selected to complement or extend the absorption properties of its binary host [17,18]. Various materials, such as small molecules, polymers, fullerene derivatives, or non-fullerene compounds, can be used as the additional third component in OPV cells [18,19]. The addition of an extra component can lead to an enhanced charge transfer and separation process for effective charge collection by electrodes [20]. In addition to the third component being able to improve light absorption, which results in a higher J_SC_, it can adjust the blend morphology at the donor–acceptor interface or provide better alignment of the energy levels, as has been highlighted in several reports [19,20,21]. However, unlike binary blends, morphology control in ternary mixtures is more difficult due to the miscibility of the components [22]. Some studies have reported that the insertion of an additional component characterized by high crystallinity can facilitate the phase separation of the binary active layer while maintaining the domain pure [22].

For a long time, fullerene and its derivatives have been the most used acceptors in the fabrication of OPV cells’ active layer due to their good carrier mobility (compared to other organic materials) and high electron affinity [23]. However, their poor absorption in the visible spectral range, high production costs, and unstable morphology limit the electrical parameters of structures based on them [24,25]. While the donor material is usually responsible for absorption in a BHJ, recently, it has been shown that non-fullerene acceptors can also be used alongside the donor to harvest light. Non-fullerene acceptors exhibit better electron mobility, tunable energy levels, and can be synthesized at low cost [10,26]. Among electron-accepting molecules, perylene diimides (PDIs) and their derivatives are attractive due to their outstanding properties, such as high electron affinity, increased electron mobility, and photochemical and thermal stability [27]. Moreover, their high absorption coefficient is another reason for the use of these compounds in OPV cell structures [28]. Also, the presence of substituents in the perylene core results in derivative compounds with improved solubility and tunable energy levels with adequate optoelectronic and self-assembling properties [29].

In this context, the aim of this work was to evaluate the properties of matrix-assisted pulsed laser evaporation (MAPLE)-deposited binary and ternary layers based on zinc phthalocyanine (ZnPc), a compound from the perylene diimide family, N,N′-bis-(1-dodecyl)perylene-3,4,9,10 tetracarboxylic diimide (AMC14), or buckminsterfullerene (C60). ZnPc (the donor) is a small-molecule compound frequently used in OPV cells due to its absorption properties and stability [30,31]. AMC14 was selected as the sole or second acceptor beside C60 due to its good solubility and absorption in the visible solar spectrum [32]. The MAPLE technique was developed to deposit soft materials using low concentrations of organic compounds (usually 1–5% mass concentration) [33,34,35,36,37]. The MAPLE process uses a frozen target containing the material (single or composite) of interest (solute) diluted in a compatible solvent (matrix), adequately selected in order to present absorption at the laser wavelength, to be volatile, and to ensure the dissolution of the organic material, etc. [35,36,37]. Firstly, an organic material–solvent mixture is prepared, then immersed in liquid nitrogen in order to obtain the frozen target. Subsequently, the target is placed into a vacuum chamber and submitted to pulsed laser irradiation, resulting in the ejection of organic molecules and solvent molecules. The organic molecules are deposited on the substrates, while the solvent molecules are pumped outside by the vacuum system. Considered a “pseudo-dry” technique [36,37], MAPLE allows the deposition of a large number of organic compounds, including biomaterials, small molecules, oligomers, polymers, etc., for a wide range of applications [33,34,35,36,37]. It has to be mentioned that in comparison to spin-coating, the most common deposition method for organic films, MAPLE presents some advantages. The spin-coating method uses concentrated solutions, a large part of which (~90%) is wasted during the deposition of the films, while the MAPLE process involves small quantities of materials [38]. Another advantage of MAPLE over spin-coating consists in the fact that substrates with different wettability can be used, with no prior treatment necessary [39]. Practically, flat or patterned substrates based on glass, plastic, transparent, conductive electrodes, etc., can be easily covered by organic films using the MAPLE technique [32]. Also, in contrast to spin-coating, where mostly orthogonal solvents must be used to obtain stacked layers (multilayer structure), in the MAPLE process, the same solvent can be used to deposit overlapping films without damaging the previously deposited layer [40]. Furthermore, MAPLE allows the deposition of thin films on various plastic substrates [41]. Nowadays, flexible electronics is emerging as a viable technology for applications in PV devices, foldable displays, wearable health-monitoring devices, and so on.

## 2. Experimental

### 2.1. Materials

**Scheme 1:**
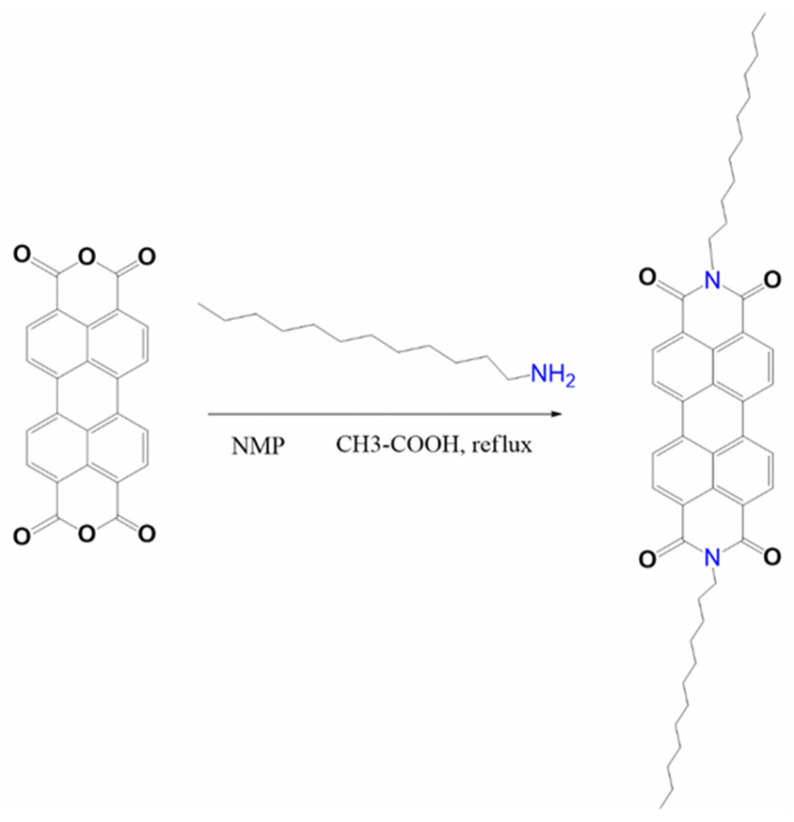
The synthesis route of the AMC14 compound.

ZnPc and C60 powders were purchased from Aldrich (Merck KGaA, Darmstadt, Germany) and Ossila (Sheffield, UK), respectively, and used without further purification. The AMC14 compound was prepared according to references [32,42], the synthesis route being given in Scheme 1. Firstly, 2 g (0.005 mol) perylene-3,4,9,10-tetracarboxylic dianhydride, 2.07 g (0.01 mol) dodecylamine, 1 mL acetic acid, and 30 mL N-methyl-2-pyrrolidone (NMP) were put into a round-bottomed flask and stirred under argon flow for 8 h at 85 °C. The mixture was cooled at room temperature, and the bordeaux-red precipitate was separated by filtration, washed with methanol, and dried in vacuum. The yield was 50%. 1H-RMN (400 MHz, CDCl_3_): δ 8.66 (d, 4H perylene), 8.60 (d, 4H perylene), 4.20 (t, 4H, N–CH_2_–), 1.77–1.80 (m, 4H, –CH_2_–), 1.25–1.47 (m, –CH_2_–), 0.88–0.91 (m, 6H, –CH_3_).

**Figure 1:**
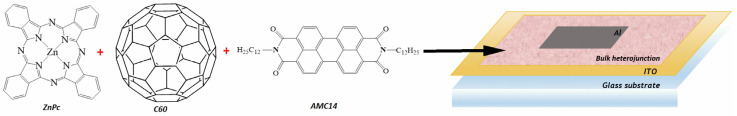
Chemical structure of the organic materials used in the MAPLE deposition and schematic representation of the structure developed with the MAPLE-deposited thin films.

ITO–glass slides 15 × 20 mm in size were bought from Ossila and used as substrates in the MAPLE deposition. The chemical structure of ZnPc, C60, and AMC14 and a schematic representation of the structure fabricated with the organic thin films deposited by MAPLE are presented in Figure 1.

### 2.2. Device Fabrication

The ITO–glass substrates were cleaned by sonication in Hellmanex solution, rinsed with deionized water, and dried with nitrogen. Prior to the MAPLE deposition, the substrates were cleaned by oxygen plasma treatment for 10 min at a pressure of 10 mbar (Electronic Diener Plasma-Surface-Technology, Pico model equipment, Plasma, Dallas, TX, USA) in order to improve the adherence of the organic films.

Thin films containing different amounts of organic compounds were deposited by MAPLE using dimethyl sulfoxide (DMSO) as solvent for the target preparation. All depositions were carried under the same experimental conditions (5 × 10^−4^ mbar pressure in the deposition chamber, 300 mJ/cm^2^ laser fluence, number of laser pulses 70,000, 5 cm target–substrate distance, 20 Hz frequency) using a KrF* excimer laser source (CompexPro 205, Lambda Physics Coherent, Gottingen, Germany) operating at λ = 248 nm and τ_FWHM_ ~25 ns.

The concentration of the solutions used to fabricate the donor–acceptor1–acceptor2 composite layers was 4.5 mg/mL with different weight ratios between ZnPc, C60, and AMC14, respectively. Reference samples containing only a single organic compound were also prepared from solutions with 4.5 mg/mL concentration. The depositions were performed at room temperature without the intentional heating of the substrate. The compositions and the labels of the prepared samples as well as their thickness and roughness are listed in Table 1. In the same MAPLE deposition process, besides ITO/glass, other substrates such as silicon and glass were deposited to obtain the samples necessary for different investigations.

The active binary or ternary layers were used to develop heterostructures with an ITO/active layer/metal configuration (Figure 1). The metallic electrode consisted of 100 nm aluminum (Al) layer deposited by vacuum thermal evaporation (1.6 × 10^−6^ mbar pressure) with a Tecuum AG (Winterthur, Switzerland), VCM600-V3-80 setup.

### 2.3. Characterization

The thickness of the fabricated layers was evaluated with an Ambios Technology XP 100 Profilometer (Ambios Technology, Inc., Santa Cruz, CA, USA). Measurements were taken on the samples (including references) deposited on glass. The thickness is given as the average value between three measurements taken at different points. The thickness of the organic layers varied between 105 nm (for ZnPc:C60) and 405 nm (for AMC14).

The vibrational and optical properties of the MAPLE-deposited thin films were analyzed as follows: (i) Fourier transform infrared spectra recorded with an IRTracer-100 spectrometer from Shimadzu, (ii) transmission spectra acquired in the 200–1000 nm range using a Carry 5000 spectrophotometer (Varian, Inc., Palo Alto, CA, USA), and (iii) photoluminescence spectra acquired at λ_exc_ = 435 nm using an FL 920 Edinburg Instruments spectrometer (Edinburgh Instruments Ltd., Livingston, UK) with a 450W Xe lamp excitation and double monochromators on both excitation and emission.

The morphology of the MAPLE organic layers was investigated with a Zeiss Merlin Compact field-emission scanning electron microscope (Zeiss, Oberkochen, Germany). The roughness of the films’ surface was evaluated with a Nanonics MultiView 4000 atomic force microscope (Nanonics, Jerusalem, Israel) working in phase feedback.

The electrical properties were investigated from the current–voltage (I–V) characteristics of the fabricated structures (ITO/mixed layer/Al) recorded in the dark and under solar simulation (AM 1.5, 100 mW/cm^2^). The experimental set-up is composed from a Keithley SourceMeter 2400 model (Tektronix, Beaverton, OR, USA), a Newport Oriel monochromator (Newport Corporation, Irvine, CA, USA), and a Newport Oriel solar simulator, operated by a computer.

## 3. Results and Discussion

### 3.1. Surface Morphology

**Figure 2:**
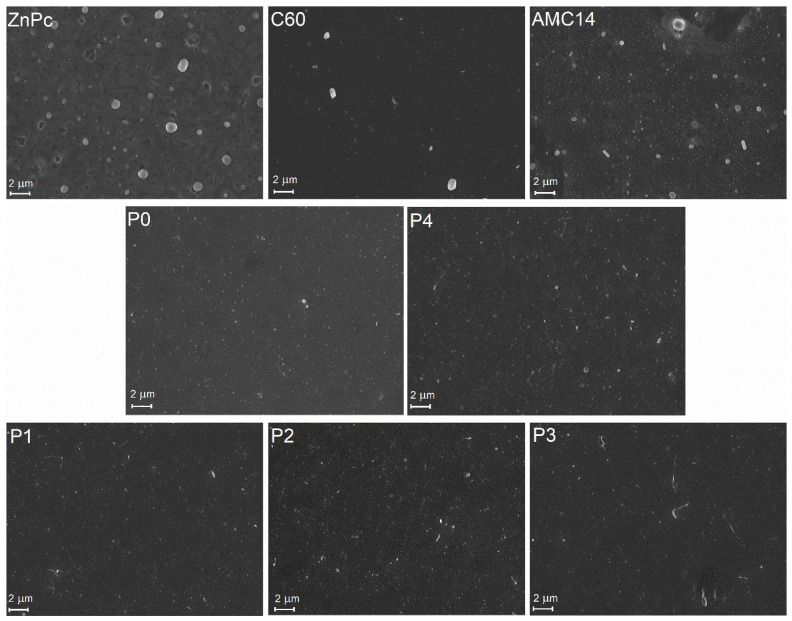
FESEM images of the MAPLE-deposited organic thin films.

**Figure 3:**
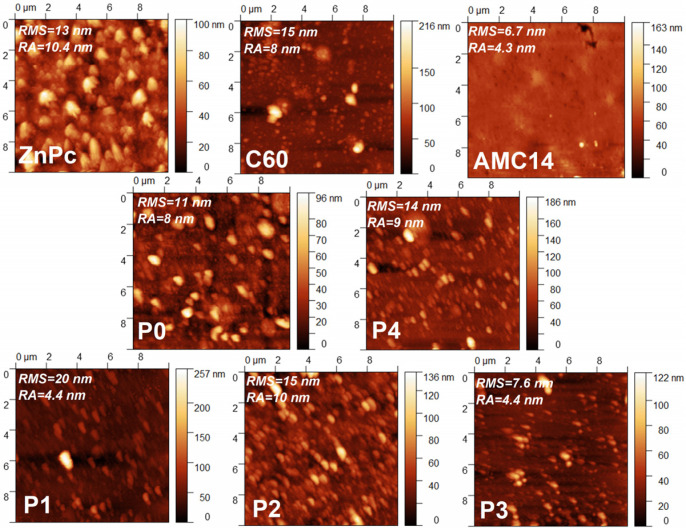
2D AFM images of the MAPLE-deposited organic layers.

The thickness and morphology (Figure 2 and Figure 3) of MAPLE binary and ternary layers were analyzed by comparison to those of the films based on a single component. The films based on the single component (ZnPc, C60, and AMC14) showed the greatest thickness (Table 1). It has to be mentioned that all MAPLE films were deposited using solutions with the same concentration (4.5 mg/mL) of the organic solute in the solvent matrix, the ratios between the three components, ZnPc, C60, and AMC14, being varied. Thus, the results can be explained taking into consideration the different displacement of the molecules on the deposition substrate depending on the ratio of the components and their molecular structure. Each component, ZnPc, C60, and AMC14 has a specific molecular structure, which is responsible for the displacement of the molecules on the deposition substrate: perylene diimide-derivative compound (AMC14) molecules tend to be arranged tilted and not parallel to the substrate surface [43], while ZnPc molecules tend to arrange parallel to the substrate surface [44]. FESEM and AFM images revealed the typical globular morphology of the organic films obtained by the MAPLE process (Figure 2 and Figure 3) [32,33,34]. However, a spongy surface was shown in the FESEM image of the ZnPc film. Moreover, the AFM image of the ZnPc film suggested that some clusters were trapped inside the film. The FESEM image of the AMC14 layer showed a morphology similar to that of the ZnPc layer than to those observed for the composite films.

The roughness values determined from the AFM measurements are not too high compared to other organic layers deposited by MAPLE or other alternative deposition methods [34,35]. It has to be noted that in the case of the single-component films, an increase in thickness led to a decrease in surface roughness, a trend that was not observed for the blended layers. The AMC14 film with the highest thickness (405 nm) was characterized by the lowest roughness value (6.7 nm). The AFM investigations suggested that a smooth surface with a reduced number of inclusions (smaller than that of ZnPc film) and characterized by a low RMS value (6.7 nm) was achieved for this acceptor. This flat surface may suggest that this PDI derivative is more soluble in DMSO than ZnPc and C60, a similar effect being previously reported for such types of compounds [45]. Concerning the other two components, it is known that fullerene is not an easily soluble molecule, while ZnPc is probably one of the most soluble metal phthalocyanines [46]. However, even though the ZnPc film presented aggregates on its surface (Figure 2 and Figure 3), they were more uniformly distributed, leading to a smaller roughness compared to the C60 films, which presented aggregates randomly distributed on their surface, resulting in an increase in roughness.

Comparing the binary layers (P0 and P4), it was noted that the films with C60 as acceptor were thinner (105 nm) and less rough (11 nm) than the film based on AMC14 (145 nm thickness and 14 nm roughness). This result suggests that inside P0 film, the C60 molecules with a diameter of ~0.7 nm was well incorporated among the ZnPc molecules with a diameter of 1.5 nm [47,48]. Moreover, the addition of C60 in the film with a higher content of AMC14 (P3) decreased both the thickness (130 nm) and roughness (7.6 nm) of the layer. The P1 sample containing the higher amount of ZnPc was the roughest (20.3 nm) and the thickest (165 nm) film from the binary or ternary mixed layers. A decrease in the roughness was achieved by lowering the ZnPc content in the P2 and P3 samples. Consequently, the thickness and roughness values of the MAPLE-deposited films were adequately for their integration in structures with applicability in the field of optoelectronic devices.

### 3.2. Optical Characterization

#### 3.2.1. FTIR Spectroscopy

**Figure 4:**
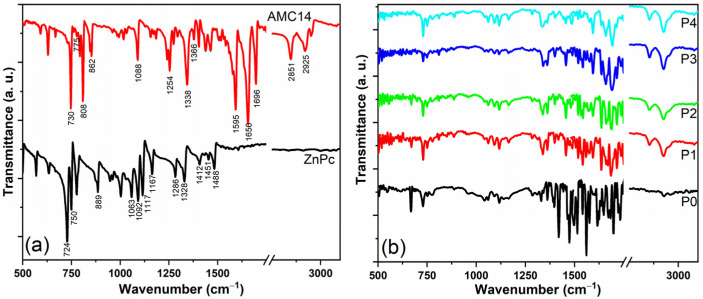
FTIR spectra of the MAPLE-deposited organic layers: (**a**) ZnPc and AMC14 thin films and (**b**) mixed thin films.

The FTIR analysis (Figure 4) was performed in order to evaluate if the chemical structures of the source organic materials were preserved during the MAPLE deposition. In the MAPLE-deposited mixed films, the specific vibrations of the functional groups from ZnPc and AMC14 compounds were revealed (Table 2). Since ZnPc and AMC14 are aromatic compounds, some specific vibrations are common to both organics. The presence of the main vibrational fingerprints confirmed that the chemical structure of the organic compounds was preserved during the MAPLE deposition.

The presence of C60 in the MAPLE-deposited films can be linked only to its characteristic peak located at ~1422 cm^−1^ [49], the peak being identified for the P0 sample, barely noticed for the P1–P3 sample, and missing in the case of the P4 sample (containing only ZnPc and AMC14). It has to be mentioned that there is a small probability that fullerene undergoes structural changes at 250 mJ/cm^2^ (the laser fluence used in this study in the MAPLE deposition), considering that C60 does not decompose at fluences below 1400 mJ/cm^2^ [50]. Also, there is a reduced possibility for C60 to interact with other molecules (from blends) due to its stability (~7.4 eV binding energy per carbon atom [50]).

**Table 2:** Assignments of the main FTIR bands of ZnPc and AMC14 [51,52,53,54].

ZnPc		AMC14	
Wavenumber (cm^−1^)	Assignment	Wavenumber (cm^−1^)	Assignment
724	C–H out-of-plane deformation	730	C–H bend
750	C–H in-plane bending	775	C–H bend
889	Isoindole stretching	808	C–H bend
1063	C–H bending	862	C–H bend
1092	C–H in-plane bending	1088	C–C bend
1117	C–H in-plane bending	1254	C–C bend
1167	C–H bending	1338	C–N stretching
1286	C–H in-plane bending	1366	C–N stretching
1328	In-plane pyrrole stretching	1595	C=C stretching in aromatic
1412	Isoindole stretching	1656	Imide C=O out-of-plane asymmetric stretching
1451	Isoindole stretching	1696	Imide C=O in-plane asymmetric stretching
1488	C=C benzene stretching	2851	C–H aliphatic stretching
		2925	C–H aromatic stretching

#### 3.2.2. UV-Vis Spectroscopy

**Figure 5:**
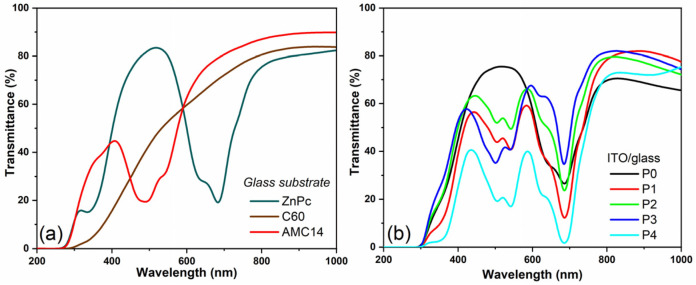
UV-Vis spectra of the MAPLE-deposited organic layers: (**a**) single thin films on glass substrates and (**b**) mixed thin films on ITO–glass substrates.

The UV-Vis spectra of the MAPLE-deposited thin films (Figure 5) were plotted to analyze the absorption properties of the (i) single (ZnPc, C60 and AMC14) layers deposited on glass and (ii) the blend films obtained on ITO/glass substrate (used as active layers in the developed structures). The ZnPc layer (Figure 5a) displayed a typical UV-Vis spectrum, with two electronic absorption domains specific to phthalocyanines. Thus, at high energy, a Soret band (under 400 nm) was visible up to around 340 nm, while at low energy, a Q band was visible between 600 nm and 800 nm. The excitonic coupling between the neighboring molecules is responsible for the Q-band split, with two maxima appearing at around 630 nm and 690 nm [55]. The non-fullerene acceptor-based film presented strong absorption in the visible region, complementary to that of ZnPc.

In the UV-Vis spectrum of AMC14, typical absorption bands characteristic of the PDIs due to the electronic transition of the individual molecules and to the π–π stacking interactions within the PDI self-assembly, with two maxima at ~490 nm (0 → 1 transition) and at ~540 nm (0 → 0 transition that usually is at ~530 nm) [56,57], can be noted. The shift to higher wavelengths is specific to the aggregated perylenimide chromophores, where interactions between the neighboring molecules takes place [58]. It is known that for this PDI class of compounds with a large exposed π surface, there is a tendency to generate aggregates when they are deposited as thin films [34,59]. Moreover, the clustering of the PDI molecules is favored by polar aprotic solvents such as DMSO [60], which was used in the present study. In comparison to ZnPc and AMC14, which showed specific intense absorption bands in the visible range, C60 revealed a low absorption with a barely noticeable weak shoulder around 450 nm, its strong characteristic absorption bands being usually identified in the UV spectral range [61,62].

The absorption bands of ZnPc and AMC14 from the visible part of the spectrum were identified in the UV-Vis spectra of the mixed layers deposited on ITO/glass substrates (Figure 5b). The bands’ intensity was related to the film thickness and the component ratio. Thus, the more intense bands were revealed by the UV-Vis spectrum of the P4 film containing ZnPc and AMC14 in equal proportion with a thickness of 145 nm. Another observation was that the intensity of the absorption maxima (from 490 nm and 540 nm) attributed to the acceptor was reversed (except for the P3 layer with the highest content of AMC14) in comparison to that of the AMC14 layer. This intensity reversal between the 0 → 0 and 0 → 1 vibronic bands is characteristic of the PDI compound and occurs when the molecules are stacked or folded on top of one another, being an aggregation indicator [58,63,64]. This may also indicate that dimers instead of oligomeric aggregates are formed [65]. Taking into consideration that the AFM measurements showed the lowest roughness for the P3 mixed film, it can be deduced that this film had the lowest degree of aggregation. The UV-Vis investigations proved that the presence of AMC14 with ZnPc can improve the light-harvesting properties due to the complementary absorption spectra.

#### 3.2.3. Photoluminescence

**Figure 6:**
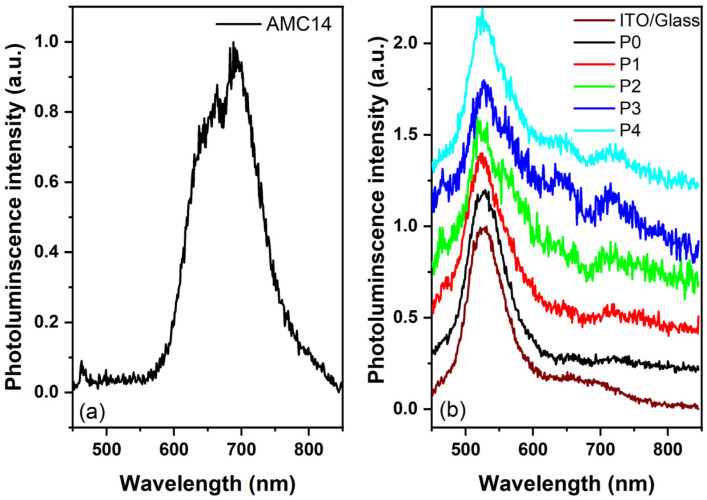
PL spectra of the MAPLE-deposited organic layers: (**a**) AMC14 thin film on Si substrate and (**b**) mixed thin films on ITO/glass substrates.

The PL spectra of the mixed films containing ZnPc, C60, and AMC14 were collected at λ_exc_ = 435 nm (Figure 6). It is known that these components can present emissions at wavelengths shorter than 600 nm (AMC14) and wavelengths longer than 600 nm (ZnPc, C60, AMC14) [32,66,67]. The PL spectrum of AMC14 layer deposited by MAPLE was also recorded on Si substrate (Figure 6) in order to avoid a possible influence of the ITO/glass substrate. The AMC14 emission spectrum shows a double-peak emission band (560–830 nm) with maxima at about 650 nm and 690 nm specific to this compound [34]. The assignment of this band and a possible explanation of the mechanisms involved are described in detail in a previous study [32]. Concerning the samples prepared on ITO/glass substrates, the AMC14 emission band was barely visible in their PL spectra, these being dominated by the emissions recorded on these commercially substrates. It has to be mentioned that some studies noted similar optical behavior for various commercial ITO/glass substrates [68,69]. Regarding the AMC14 emission band, this was better highlighted in the emission spectra of the layers with higher AMC14-acceptor content, P3 and P4 samples. In the samples containing both C60 and AMC14 acceptors, the intensity of this peak increased with the increase in AMC14 content from P1 to P3. As in the case of the UV-Vis spectra, a shifted towards higher wavelengths of the emission maximum was noted in the PL spectra, suggesting interactions between the chromophores of the neighboring molecules. The relative orientation of the molecules strongly influenced the magnitude of the interaction (coupling). Moreover, the red shift of the PL maximum suggested the presence of H-aggregates with chromophore transition dipoles, with their long axes disposed parallel [59].

A quenching effect of the AMC14 emission band took place after blending this component with the other organic compounds. As already reported for PDI derivatives, the aggregation process can also induce emission quenching due to the charge transport, which occurs along the π–π stacking direction. Practically, the quenching mechanism is favored because the photogenerated carriers are spatially separated and the recombination is reduced [56,70]. The energy transfer from the photoexcited PDI acceptor to the ground state of the donor material can be another possible explanation for the PL quenching in the investigated blends. Also, the charge transfer from the acceptor to the donor can lead to the extinction of the emission. The quenching effect may also depend on the film thickness and materials’ ratio [71].

### 3.3. Electrical Measurements

**Figure 7:**
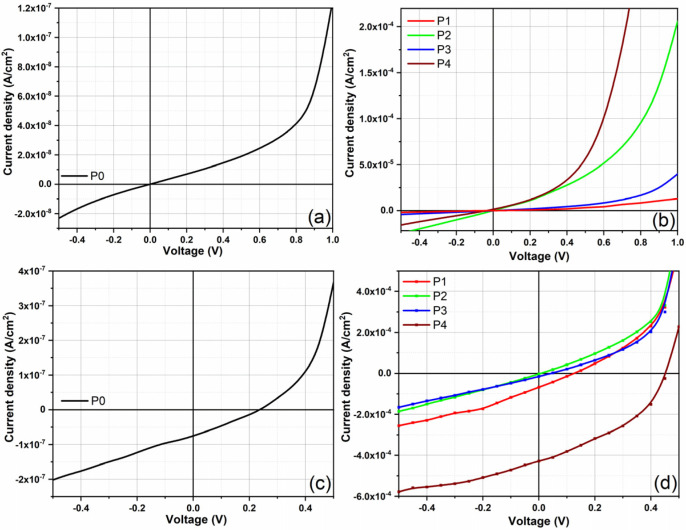
J-V characteristics, plotted in the dark (**a**,**b**) and under illumination (**c**,**d**), of the structures prepared with the MAPLE-deposited organic films.

The J-V characteristics were acquired both in the dark and under illumination. The fabricated structures showed rectifying properties resulting from the shape of the characteristics plotted in the dark (Figure 7a,b). In addition to the energy-level alignment, the component ratio, and the layer morphology, the microstructure of the blend has a major contribution to the current density values [34,72]. By adding the perylene diimide acceptor as the third component, an increase in the current density values was firstly observed in comparison to that obtained for the P0-based structure containing only C60 (1.9 × 10^−8^ A/cm^2^) at 0.5 V applied voltage. This increase can be correlated with the AMC14 ratio (3.2 × 10^−6^ A/cm^2^ for P1 with 25% AMC14 and 3.8 × 10^−5^ A/cm^2^ for P2 with 33% AMC14). Thus, apart from C60 acting as an acceptor, the AMC14 can also influence to some extent the transport and collection of charges. Surprisingly, the structure based on the P3 layer showed low current density (5.9 × 10^−6^ A/cm^2^) even though the P3 layer was the smallest roughness (RMS = 7.6 nm), which means that not only the morphology is responsible for the decrease in current.

From the point of view of energetic barriers (Table 3), HOMO levels of the implied materials (ZnPc, C60, and AMC14) were placed to facilitate the hole transport. AMC14 induced a lowering in the dark current when its ratio surpassed that of the first acceptor. In terms of charge generation, a parallel-like model where the third compound acts as an individual electron-transport channel with no energy or charge transfer between the proposed acceptors (C60 and AMC14) [19] can be assumed. Moreover, limitations related to the energy level position are not imposed if the driving force between the donor (ZnPc) and the first acceptor (C60) is sufficient to dissociate the generated excitons [73].

Thus, neither morphology nor energetic barriers are determinant factors affecting charge carrier transport. The reason for the current density drop could be the miscibility between ZnPc, AMC14 (in a significant proportion), and C60, the microstructure of the blend not being favorable for charge carrier transport. Probably, the insertion of a large amount of AMC14 beside C60 introduced a high degree of disorder, which affected the charge carrier movement. The higher dark current density value (5.3 × 10^−5^ A/cm^2^) was recorded in the structure based on P4 film with the total replacement of C60 with AMC14.

The current density in P4 with AMC14 only was 5.3 × 10^−5^ A/cm^2^, while that in P0 containing only C60 was 1.9 × 10^−8^ A/cm^2^, despite the greater thickness of the P4 film (145 nm) compared to the P0 film (105 nm), which means that carriers must cross more interfaces and travel longer distances to be collected at the electrodes. The dark current is the result of the carrier generation and recombination at different interfaces. The dark current of such structures is determined by the carrier mobility in the constituent materials. In reference [77], it is mentioned that the mobility of PDI derivatives can be up to 10x higher than the most used fullerene derivatives in OPV structures. Therefore, it can be assumed that the high dark current is due to the higher carrier mobility of AMC14 in P4 compared to C60 in P0 [78]. Even if the LUMO position in the AMC14 (E_LUMO_ = 3.44 eV [76]) indicates that it can favor trap-limited electron transport [79], the current density values obtained in structures P1–P4 suggest that these traps can act mostly in the case of the P3 sample. Also, a trap-limited hole transport can appear in the case of structures P0–P3 prepared with C60 due to the low position in the energy scale of HOMO level in C60. The major influence of the hole-trapping mechanism on the current was revealed by sample P0 containing ZnPc and C60 blended at a weight ratio of 1:1. The difference obtained between the dark current values recorded for the P0 and P4 samples suggests that these capture states can play an important role in the hole transport in sample P0.

The J-V characteristics recorded under illumination for the fabricated structures are displayed in Figure 7c,d and the electrical parameters extracted from these characteristics are presented in Table 4. Compared to the P0-based structure (J_SC_ = 7.5 × 10^−8^ A/cm^2^, V_OC_ = 0.24 V), an increase in J_SC_ value (J_SC_ = 6.7 × 10^−5^ A/cm^2^) with a decrease in V_OC_ (0.12 V) were obtained for the structure based on P1. Because the P1 film was thicker (170 nm), a better absorption in the active layer can be anticipated, but at the same time, an increase in the carrier path to the electrodes and thus in the recombination probability can also occur. Thus, the J_SC_ was associated with the improved light harvesting (by adding AMC14 in the blend), while the charge recombination was probably responsible for the lowering of the V_OC_ value. Usually, in organic cells, the V_OC_ value is given by the difference between the HOMO level of the donor and the LUMO level of the acceptor, but according to the literature, various parameters such as the interface area between donor and acceptor, the active layer morphology, the defect states, crystallinity of the prepared layers, etc., [80,81] can influence the V_OC_ value. The higher thickness of the P1 compared to P0 and P4 can be associated with an increased number of grain boundaries, which favors the recombination of the separated charges.

Increasing the PDI derivative content (P2 and P3 samples), the process of carrier photogeneration becomes very weak. As already mentioned, the microstructure of the blend is important, the presence of C60 affecting the molecular packing inside the film. Thus, efficient collection of the charges at the electrodes was not evidenced for the structure fabricated with the film containing higher C60 content (P2 sample). On the other hand, P3 with a lower C60 content was thinner than the P2 film, which means reduced absorption, but at the same time, reduced recombination on the boundaries. This allows the transport and collection of a certain number of charge carriers, this process generating the weak photovoltaic effect found.

An increase in the electrical parameters was obtained using perylene diimide derivative instead C60, probably due to better light harvesting by using this acceptor with good absorption in the visible domain compared to that of the fullerene. Although the values obtained in our study are smaller than or similar to those reported in the literature for OPV cells prepared with other PDI acceptors [82,83,84,85], an enhancement can be further achieved by: (i) using different ETL and HTL buffer layers (ZnO, PEDOT:PSS, MoO_3_, etc.) in order to facilitate the collection of the charge carriers to the electrodes, (ii) selecting a proper solvent or using different additives in order to obtain an intimate contact between the blend constituents and thus an adequate morphology, (iii) and using light-trapping schemes (e.g., employing nanostructured electrodes or inserting metallic nanoparticles in the organic film) to improve the harvesting process in the active layer.

## 4. Conclusions

A new perylene diimide derivative compound was used to gradually replace (until total replacement) a C60 fullerene acceptor in mixed films based on a ZnPc donor. The investigations revealed that the composite films deposited by MAPLE presented the particularities of both metal phthalocyanine and new acceptors inserted in the blends. The morphology of the layers is characteristic of the MAPLE deposition process being influenced by the composition of the layers. The film’s surface had reduced roughness compared to other films deposited in similar conditions using DMSO as solvent. The FTIR spectra recorded on the prepared films revealed only the distinct vibrations of ZnPc and AMC14 compounds. The UV-Vis spectra clearly showed absorption bands attributed to ZnPc (donor) and AMC14 (acceptor), demonstrating that this PDI derivative compound can be used to improve light harvesting. The emission band associated with AMC14 was quenched when this compound was mixed with ZnPc and C60.

A dark current density of about 5.3 × 10^−5^ A/cm^2^ (at 0.5 V applied voltage) was obtained for the structure prepared with P4 film, where C60 was totally replaced by the AMC14 acceptor. Also, the J-V characteristics plotted under illumination showed an increase in J_SC_ value, even when a small amount of PDI derivative replaced C60 (P1 sample) compared to the structure based on the ZnPc:C60 layer, most probably due to better harvesting of light. This result evidences the potential of such perylene diimide derivative compounds to act as acceptors in photovoltaic structures.

## Figures and Tables

**Table 1:** Composition, label, thickness, and roughness values of the MAPLE thin films.

Sample	Component Ratio	Label	Thickness (nm)	RMS (nm)	R_a_ (nm)
ZnPc	-	ZnPc	295	13.7	10.7
C60	-	C60	210	15.4	8.6
AMC14	-	AMC14	405	6.7	4.3
ZnPc:C60	1:1	P0	105	11.0	7.9
ZnPc:C60:AMC14	2:1:1	P1	170	20.3	12.8
ZnPc:C60:AMC14	1:1:1	P2	150	15.0	10.7
ZnPc:C60:AMC14	1:1:2	P3	130	7.6	4.4
ZnPc:AMC14	1:1	P4	145	14.0	9

**Table 3:** Highest occupied molecular orbital (HOMO), lowest unoccupied molecular orbital (LUMO), bandgap, and absorption peaks of the constituent organic compounds.

Compound	HOMO	LUMO	Bandgap	Absorption Peaks
ZnPc	5.17 [74]	3.78 eV [74]	~1.4	340, 630, 690 [55]
C60	6.2 [75]	3.7 eV [75]	~2.5	340, 440 [62]
AMC14	5.16 [76]	3.44 eV [76]	~1.7	490, 530 [56,57]

**Table 4:** Comparative parameters of the structures fabricated with MAPLE-deposited mixed layers based on ZnPc, C60, and AMC14.

Sample	V_OC_ (V)	J_SC_ (A/cm^2^)	P_max_ (W/cm^2^)	FF	PCE (%)
P0	0.24	7.5 × 10^−8^	0.3 × 10^−8^	0.19	0.34 × 10^−5^
P1	0.12	6.7 × 10^−5^	0.2 × 10^−5^	0.28	0.22 × 10^−2^
P4	0.46	4.3 × 10^−4^	0.7 × 10^−4^	0.35	0.7

## Data Availability

The original contributions presented in the study are included in the article, further inquiries can be directed to the corresponding author.
